# Adaptation and Acclimation of Gametophytic Traits to Heat Stress in a Widely Distributed Wild Plant Along a Steep Climatic Gradient

**DOI:** 10.1002/ece3.71199

**Published:** 2025-03-30

**Authors:** Donam Tushabe, Franziska Altmann, Erik Koehler, Sebastian Woods, Sandra Kahl, Sergey Rosbakh

**Affiliations:** ^1^ Ecology and Conservation Biology, Institute of Plant Sciences University of Regensburg Regensburg Germany; ^2^ Biodiversity Research/Systematic Botany, Institute of Biochemistry Und Biology University of Potsdam Potsdam Germany; ^3^ Department of Plant and Environmental Sciences, Faculty of Science University of Copenhagen Frederiksberg Denmark

**Keywords:** acclimation, climate change, climatic gradient, gametophytes, heat waves, local adaptation, seed, *Silene vulgaris*

## Abstract

Climate change‐induced heat waves often reduce seed yields and quality via high‐temperature effects in the gametophytic phase. Yet, in contrast to model and crop species, the ability of pollen and ovules to adapt or acclimate to heat stress in wild plants remains poorly understood. To address this gap, we examined the adaptation and acclimation potential of six gametophytic traits in 11 wild *Silene vulgaris* populations across a temperature gradient in Europe. First, we cultivated plants in a common garden to reveal differences in gametophytic traits indicative of adaptation. Next, we assessed their acclimation potential by subjecting flowering plants to two chronic heat stress (CHS) treatments: moderate (35°C/30°C) and severe (40°C/35°C) for 18 days. Also, we estimated the CHS effects on seed quantity and quality. The common garden experiment showed no intraspecific variation in gametophytic traits across the temperature gradient, suggesting these traits may not influence reproductive adaptation to local habitats. During CHS, the female gametophyte was less temperature‐sensitive than the male. Moderate CHS led to larger ovaries with more large‐sized ovules, while severe CHS reduced ovule numbers but increased their size. Both CHS treatments decreased pollen grain numbers, size, and anther length, with severe CHS causing greater reductions. These reductions in gametophytic traits led to lower seed yield and quality. Under both CHS treatments, acclimation potential did not vary along the temperature gradient, except for pollen size under severe CHS, which was larger in warmer climates. Our findings revealed the lack of adaptation and acclimation mechanisms in the gametophytic traits (except for pollen size) of wild *Silene vulgaris* populations along the temperature gradient. These findings suggest that *Silene* plants may rely on alternative strategies, such as shifts in gametophyte physiology and biochemistry or flowering phenology, to respond to thermal stress associated with heat waves.

## Graphical abstract

Climate change‐induced heat stress reduces seed yield and quality by affecting gametophytic traits, yet their ability to adapt or acclimate remains poorly understood. Our study on wild *Silene vulgaris* populations across a European temperature gradient found no intraspecific variation in gametophytic traits, except for pollen size under severe heat stress. These findings suggest *S. vulgaris* may rely on alternative strategies, such as shifts in flowering time, to cope with rising temperatures.

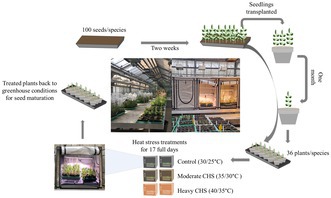

## 1 Introduction

In the face of rising global temperatures, heat waves have become increasingly severe and frequent, impacting the growth and development of plants (Hatfield and Prueger 2015). The sensitivity of crucial stages in plant development, such as the gametophytic phase, to heat stress can negatively affect reproductive processes (Hedhly 2011; Sinha et al. 2021; Yadav et al. 2022). Particularly, the heat‐wave effects on pistils, anthers, and therefore, ovules and pollen grains, often lead to diminished seed production and compromised quality (Hatfield and Prueger 2015; Raza et al. 2019; Tushabe et al. 2023). Consequently, the altered reproductive performance not only jeopardizes food security by causing significant reductions in crop yields (Kumar 2016; Seppelt et al. 2022) but also carries ecological consequences. As for the former, estimated global crop yields are expected to reduce by 10% by 2050 (Wing et al. 2021). As for the latter, altered seed production might cause declines in plant population sizes and disrupt plant–animal interaction across various trophic levels associated with natural populations (Willis et al. 2008; Bogdziewicz et al. 2016).

The ability of species to endure the consequences of heat waves mainly relies on either migration to suitable habitats or local adaptation (Pecl et al. 2017; Åkesson et al. 2021). In most cases, migration is not a viable option due to extensive seed dispersal distances (Howe and Smallwood 1982; Ellis 2015) or lack of suitable new habitats (Rumpf et al. 2018). In this regard, local adaptation and acclimation emerge as crucial survival strategies (Kleine et al. 2021; Wadgymar et al. 2022). While local adaptation involves genetic changes over generations to better suit specific environments (Rehfeldt et al. 2002; Savolainen et al. 2007), acclimation entails short‐term physiological adjustments to cope with immediate environmental changes within a plant's lifetime (Kleine et al. 2021). Both local adaptation and acclimation can enhance heat tolerance through various plant traits. These include maintaining essential physiological processes (e.g., efficient water use or improved photosynthetic mechanisms (Vincent et al. 2020)), phenotypic traits (e.g., early or delayed flowering and or changes in leaf and root morphology (Cook et al. 2012)), and genetic adaptations (e.g., increased production of heat shock proteins (Hasanuzzaman et al. 2013)).

As for plant sexual reproduction, previous research has shown that species and populations from climates experiencing frequent and/or severe heat waves tend to have higher heat tolerance of their gametophytic traits (Lancaster and Humphreys 2020; McDonald et al. 2023; Tushabe and Rosbakh 2025). Notably, these studies suggest varying tolerance levels, with species from colder regions showing greater cold tolerance and those from warmer areas displaying higher thermal tolerance (Rosbakh and Poschlod 2016; Lancaster and Humphreys 2020). Yet, the contribution and relative importance of adaptation and acclimation in plant gametophytic responses to heat stress still remain poorly understood. To begin with, existing studies have mainly focused on estimating intra‐ and interspecific variation of gametophytic traits related to heat tolerance across complex ecological gradients (Di Biase et al. 2021; Kang et al. 2022; Amimi et al. 2023). Although such studies inform us about the potential range of plant gametophytic heat tolerance, there is often a challenge in distinguishing the relative contribution of local adaptation and acclimation, because plants usually grow under field conditions that are difficult to control. This limitation can be lifted by cultivating plants representing different populations and species under controlled conditions (i.e., common garden experiment; Kahl et al. 2019; Tushabe et al. 2023), yet such experimental research has been limited either to cultivated species (see e.g., Hedhly 2011) or indoor‐grown model species (e.g., *Arabidopsis thaliana* ; Scheepens et al. 2018). Importantly, only a few of them have considered ovule and pollen traits (e.g., Flores‐Rentería et al. 2018), with most of the studies focusing on flowering phenology (e.g., Scheepens and Stöcklin 2013; Arnold et al. 2022) and/or seed traits (e.g., Zhou et al. 2021; Amimi et al. 2023). As a result, our understanding of adaptation and acclimation of gametophytic traits, which are the most sensitive to temperature stress (Hedhly 2011), in wild plant populations is still lacking.

Here, we close these gaps by testing the adaptation and acclimation potential of six gametophytic traits measured to heat stress in 11 populations of wild *Silene vulgaris* (bladder campion, Caryophyllaceae), occurring along a steep climatic gradient of temperature in Europe. In the first part of our experiment, we cultivated plants in a common garden, to reveal potential adaptations in the gametophytic traits to the local growing conditions. We hypothesized that plants originating from colder climates tend to produce smaller anthers and ovaries with fewer and smaller‐sized pollen and ovules, respectively (*H*_1_). The relatively smaller investment into gametophytic tissues of cold‐adapted plants should be therefore reflected in lower seed production, both in terms of size and number, compared to their counterparts from warmer climates. This hypothesis is based on the assumption that plant regeneration is constrained in cold environments due to the resource‐intensive nature of flowers, gametophytes, and seeds, acting as resource sinks (e.g., Obeso 2002). An equally plausible alternative hypothesis is that plants produce fewer, but larger seeds.

Next, we investigated the acclimation potential of the same gametophytic traits across the study populations by exposing the flowering plants to experimental chronic heat stress (CHS) treatments: moderate (35°C/30°C) and severe (40°C/35°C), both lasting 18 days. The moderate CHS treatment represents typical heat waves that plants, especially those in the southern regions, may have already encountered (Table 1; Lhotka and Kyselý 2022). In contrast, the severe stress represents potential future heat waves that the plants may not have acclimated or adapted to yet (Lin et al. 2022). In response to moderate CHS treatment (35°C/30°C), we expected that gametophytic traits in plant populations from warmer climates, experiencing more frequent heat waves, would exhibit a better ability to acclimate (*H*_*2*_). This is attributed to the presence of acclimation mechanisms developed in response to previous encounters with similar temperatures (e.g., McDonald et al. 2023), enabling these plants to sustain the production of normal‐sized anthers, ovaries, pollen, and ovules, resulting in unaltered seed production. Conversely, in plants from colder climates where heat waves are less common, we expected a reduced capacity for acclimation in gametophytic traits due to the lack of previous exposure to such temperatures and the consequent lack of pre‐acclimation mechanisms (Nievola et al. 2017). As a result, these plants might produce smaller anthers and ovaries containing fewer and smaller‐sized pollen and ovules, respectively, potentially resulting in altered seed production and size.

**TABLE 1:** Origins of the 11 European *Silene vulgaris* populations, including the number of individuals per population (*n*), the elevation of the sampling sites, the mean temperature during the flowering period, the mean number of days with temperatures at or exceeding 35°C and 40°C, the months of the flowering period, and the mean annual temperatures in their respective locations. The climatic data are means from 2000 to 2020, derived from the European climatic database. The flowering period data are sourced from GBIF occurrence records.

Population	Number of individuals	Country	Site	Latitude	Longitude	Elevation (m)	Nature of site	Mean annual precipitation (mm)	Potential evapo‐transpiration (mm)	Aridity index	Mean temperature of flowering period (°C)	Mean days ≥ 35 (°C)	Mean days ≥ 40 (°C)	Flowering period (months)	Mean annual temperature (°C)
SE80	24	Spain	La Palma	28°46′58.0″ N	17°56′22.0″ W	1100	Forest	432	1200	0.4	18	0	0	April–June	14.5
SE83	12	France	La Noue du Bourg	46°39′28.0″ N	01°23′13.6″ W	73	Agricultural	770	900	0.9	20	2	0	April–June	12
SE84	12	Sweden	Vickleby	56°34′37.1″ N	16°27′39.5″ E	51	Grassland	509	700	0.7	20.5	0	0	June–August	7.6
SE49	12	Spain	La Gueria Carrocera	43°19′02.0″ N	5°39′45.6″ W	348	Forest	950	1100	0.9	21.3	0	0	May–July	11.4
SE86	12	France	Normandie	49°16′06.0″ N	01°37′39.0″ E	83	Agricultural	838	850	1	22.2	3	1	May–July	10.3
SE91	13	Germany	Konstanz	47°40′42.5″ N	9°10′30.0″ E	400	Urban	879	950	0.9	24	4	0	May–July	9.2
SE35	10	Spain	Madrid	40°24′55.0″ N	3°42′45.2″ W	621	Urban	450	1300	0.4	24.5	28	2	April–June	13.8
SE31	11	Czech	Brno	49°11′45.4″ N	16°36′31.8″ E	209	Urban	550	950	0.6	24.7	4	0	May–July	8.4
SE72	10	Spain	La Sentiu de Sió	41°48′18.7″ N	0°52′43.6″ E	316	Agricultural	490	1200	0.4	25	22	3	April–June	14.4
SE98	10	Austria	Innsbruck	47°16′14.6″ N	11°24′39.7″ E	557	Urban	1100	900	1.2	26	3	0	June–August	5.7
SE82	23	Spain	Alcanó	41°29′29.4″ N	00°26′27.3″ E	211	Agricultural	366	1100	0.3	26	25	2	April–June	15

Under severe CHS treatment (40°C/35°C), we hypothesized that all populations would show reduced acclimation potential (*H*_3_). The rarity of exposure to such extreme stress conditions in these plants hinders the development of acclimation mechanisms. Moreover, the physiological constraints imposed by temperatures at 40°C, including the disruption of cellular membranes, protein denaturation, oxidative stress, and impaired gene regulation, further contribute to the inability of plants to acclimate to such extreme temperatures (e.g., Araújo et al. 2013; Hasanuzzaman et al. 2013). This is expected to have negative impacts on gametophytic traits, such as reduced pollen and ovule production, ultimately resulting in reduced seed quantity and quality.

## 2 Methods and Materials

### 2.1 Study Species and Experimental Setup

*Silene vulgaris* (Moench) Garcke (Caryophyllaceae), commonly known as bladder campion, is a short‐lived herbaceous perennial plant widely distributed across diverse geographical regions, ranging from subarctic to temperate zones (Rabinowitz et al. 1986). It is indigenous to Europe, Asia, and North Africa, with a global presence due to introductions in different regions (USDA NRCS 2023). Its primary habitats include open grasslands, meadows, woodland edges, as well as disturbed areas such as roadsides, waste places, and metalliferous soils (Marsden‐Jones and Turrill 1957; Friedrich 1979). The plant exhibits a clump‐forming growth habit, with erect or sprawling stems that can reach heights of up to 60 cm.

The genus *Silene* has been widely utilized in ecological research due to its favorable attributes, including ease of cultivation and short life cycles, facilitating experimental investigations (Bernasconi et al. 2009; Blavet et al. 2011; Kahl et al. 2019). *Silene vulgaris* particularly offers a distinctive opportunity to investigate how wild plants locally adapt and vary along thermal gradients (e.g., Kahl et al. 2019).

**FIGURE 1:**
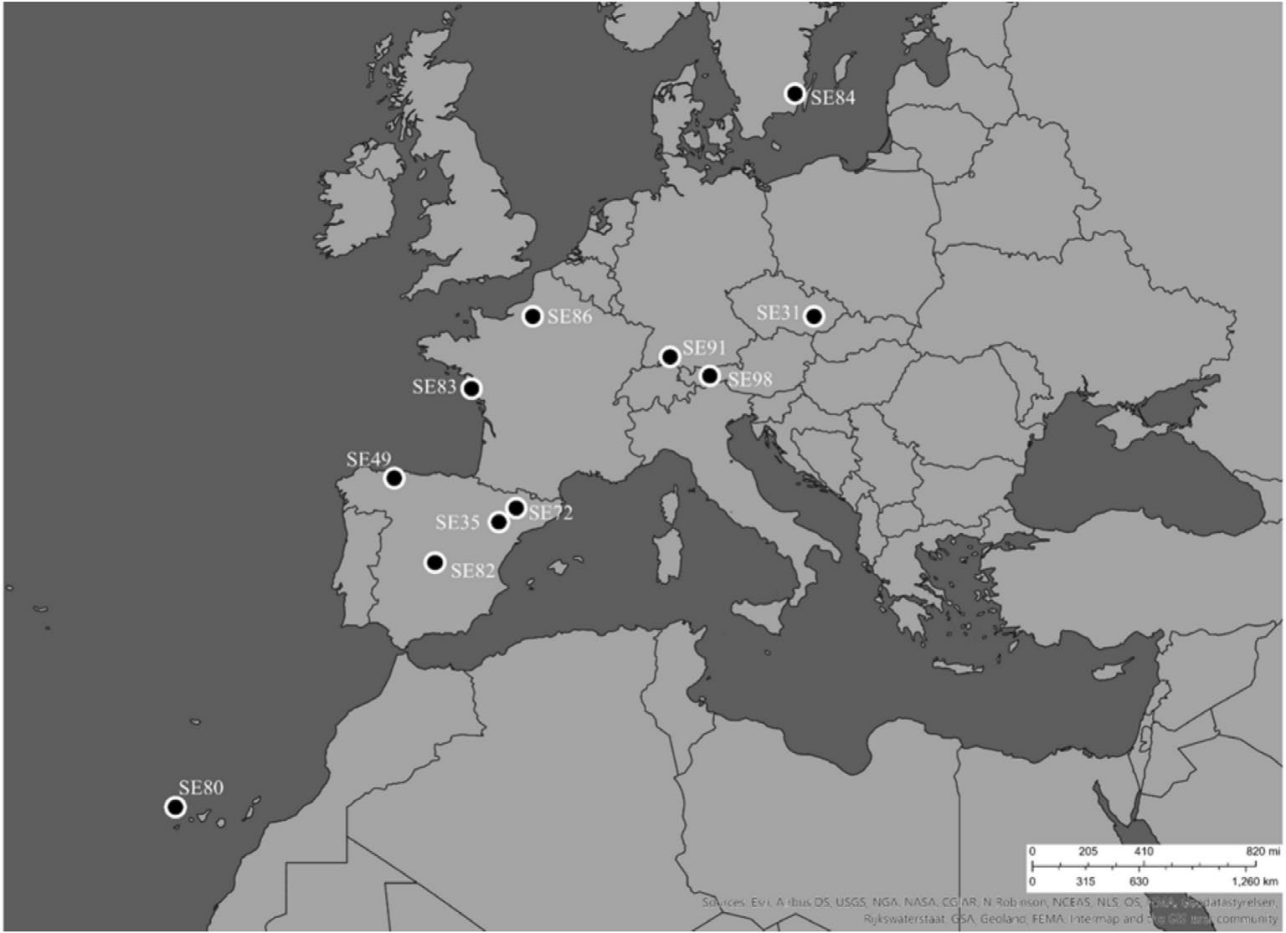
Sampling sites of the 11 *Silene vulgaris* populations across six different European countries. Map created with Esri ArcGIS Online.

The seeds were collected from 11 populations of *Silene vulgaris* across locations in Austria, Czech Republic, Germany, France, Spain, and Sweden (see Table 1, Figure 1). These populations belong to three distinct genetic clusters (see Kahl et al. 2021). Seeds were collected in 2015–2019 from at least 10 open‐pollinated plants at each site, with at least 1‐m spacing between plants. The exact locations of the sampling sites were recorded using a GARMIN GPS 72H device (accuracy < 15 m; Garmin, Switzerland). These populations represent a climatic gradient of *S. vulgaris* in Europe, which may exhibit plant sexual reproduction adaptations to varying temperature conditions. All selected populations exhibit hermaphroditic flowers, containing both male and female reproductive organs within a single flower, and a short seed‐to‐maturity period of about 7 weeks.

The experimental setup and procedures described in this study were adapted from a previous study by Tushabe et al. (2023), conducted within the same greenhouse facility from November 2022 to July 2023.

To eliminate potential maternal effects, we cultivated F1 plants from the seeds collected in the field from each *S. vulgaris* population. These plants were grown in trays filled with a substrate mixture composed of low‐nutrient planting soil, coarse sand, and dry compost soil. The plants from distinct populations were bagged before the onset of flowering to prevent cross‐pollination. Thereafter, the F1 seeds were planted in a similar soil substrate as described above. Approximately 3 weeks after germination, the young plants were individually transplanted into pots, with careful selection based on health and phenological stage to maintain consistency across treatments.

Throughout cultivation, all plants received equal attention, including random rearrangement within the greenhouse and consistent watering. The greenhouse served as a common garden, providing similar conditions (day/night temperatures of 20°C/15°C and natural illumination) for investigating the adaptation of gametophytic traits to local growing conditions. Additional lighting was employed to maintain a 12‐h photoperiod during winter months (November to April).

After the emergence of the initial flower bud, plants at a similar phenological stage were randomly selected for the CHS experiment.

The CHS experiment was conducted in six identical grow chambers (Homebox Vista Medium, HOMEbox, Germany, see Figure S1) equipped with heating mats and LED lamps for temperature and illumination control. Two chambers were maintained at day/night temperatures of 35°C/30°C, representing moderate CHS, while another two chambers were set at 40°C/35°C, indicating severe CHS. Two chambers at 20°C/15°C served as the control group (see Figure S2). The moderate and severe CHS chambers were used to investigate the acclimation potential of the gametophytic traits, aligning with heat waves currently being observed in European regions and those anticipated in the future (Lin et al. 2022). In each chamber, 10–24 plants per population were cultivated for 18 days, ensuring stress treatment at the different developmental stages of pollen and flowers (Mesihovic et al. 2016). To prevent drought stress linked to elevated temperatures, we maintained a consistent soil moisture level by regular watering during the application of heat stress. Post‐heat treatment, plants were returned to the greenhouse for seed maturation under controlled conditions.

During cultivation, only the plants in the control group had an aphid infestation and therefore received treatment with Karate zeon (Karate Zeon, Syngenta, Switzerland). The heat‐treated groups were not sprayed with Karate Zeon as they showed no aphid infestation, likely due to the environmental conditions. Plants were monitored post‐treatment, and no visible effects on measured traits were observed.

### 2.2 Measurement of Plant Traits

To evaluate the effects of the experimental treatments on overall plant performance, we conducted measurements of leaf chlorophyll fluorescence, specifically assessing the maximum quantum yield (*Fv/fm*) of PS II photochemistry. These measurements were conducted over a 20‐min duration using a Pocket PEA tool (Hansatech, Germany) on the final day (day 18) of the treatment period.

### 2.3 Gametophytic Traits

The approach used for gametophytic trait measurements in this study was similar to previous work by Tushabe et al. (2023). Six traits related to male (anther length, pollen production, and size) and female (ovary length, ovule production, and size) gametophytic performance were assessed to investigate the impact of heat treatment on sexual reproduction.

### 2.4 Seed Traits

To evaluate the subsequent impacts of heat stress on gametophytic performance, we measured seed mass and seed production in the treated plants. To accomplish this, the treated plants were kept in the greenhouse for approximately 3 months until complete seed maturation. During this period, the plants were self‐hand‐pollinated to ensure the reproductive success of the plants. Additionally, the plants were covered with organza bags to prevent any loss of seeds. Due to low seed production, it was not feasible to collect seeds from individual plants. Instead, all seeds from each accession were combined, and the corresponding seed mass and number were determined. The low seed number may be attributed to limitations in pollination, despite the plants being self‐compatible and having undergone hand‐pollination.

### 2.5 Data Analysis

#### 2.5.1 Temperature Conditions in Seed Collection Sites

Data analyses were conducted using R software version 4.3.0 (R Core Team 2023). To characterize the temperature conditions that each study population experiences during fertilization across the sampled gradient, we calculated the average temperature of the flowering period and identified occurrences of heat waves. To begin with, we extracted species occurrence data per population from the Global Biodiversity Information Facility (GBIF). Our initial assumption was that the GBIF occurrence data were derived from images and herbarium specimens of flowering individuals. Visual inspection of randomly selected images and digitalized herbarium specimens confirmed that assumption at the subsequent stage. Only human observations and preserved specimens with georeferenced locations from the years 2000 to 2020 were considered. This time frame was chosen due to the relatively short lifespan of *Silene vulgaris* , making it more relevant to gather information during the 21st century when significant global climate changes have occurred. The data included details such as the year, month, and days of observation. The flowering period was determined as the month with the highest number of observations, along with the month prior and after it (a total of 3 months).

Based on the flowering phenology data, we obtained the maximum temperature values for the respective locations within the period from 2000 to 2020. This extraction was performed using the R package *easy climate* (Cruz‐Alonso et al. 2023), which relies on high‐resolution (1 km) daily climate data sourced from the European climatic database. The mean temperature of the flowering period (MTFP) was computed based on temperatures recorded in the month with the highest number of observations, along with the month prior and after it.

To determine the occurrence of heat waves, we calculated the mean number of days with temperatures at or exceeding 35°C and 40°C per accession at the corresponding locations over the period from 2000 to 2020.

#### 2.5.2 Statistical Analysis

To estimate the variability of local adaptation and acclimation potential of the gametophytic traits within study populations across the climatic gradient, we fitted a linear mixed‐effects model using the package *lme4* (Bates et al. 2015). The model used one of the measured traits (e.g., chlorophyll fluorescence, gametophyte, or seed trait) as the response variable. The fixed effects included the MTFP, moderate and severe CHS treatments, the control temperature, and their interaction (MTFP*Treatments). To account for individual variability in the response of gametophytic traits, the origin of every plant population accession was included in all models as a random intercept. We visualized how the relationship between MTFP and gametophytic traits varies across the different heat stress treatments and the control with an interaction plot created using the *interactions* package in R (Long 2019). Local adaptation was assessed by examining the variability in the control regression slope across the temperature gradient (MTFP). Acclimation was determined by considering both the overall treatment effects and the differences in regression lines between the control and CHS treatments.

Differences in local adaptation and acclimation of gametophytic traits among plant groups from different thermal environments were assessed utilizing the post hoc Tukey test (*p* < 0.05), as applied in the *emmeans* and *multcomp* packages (Hothorn et al. 2008; Lenth 2023).

The chamber effect was not included in the model because trays were randomly rotated across chambers during trait measurements to minimize any chamber effects. Additionally, temperature and light conditions were carefully controlled to further reduce potential variation.

## 3 Results

### 3.1 Leaf Chlorophyll Fluorescence

**FIGURE 2:**
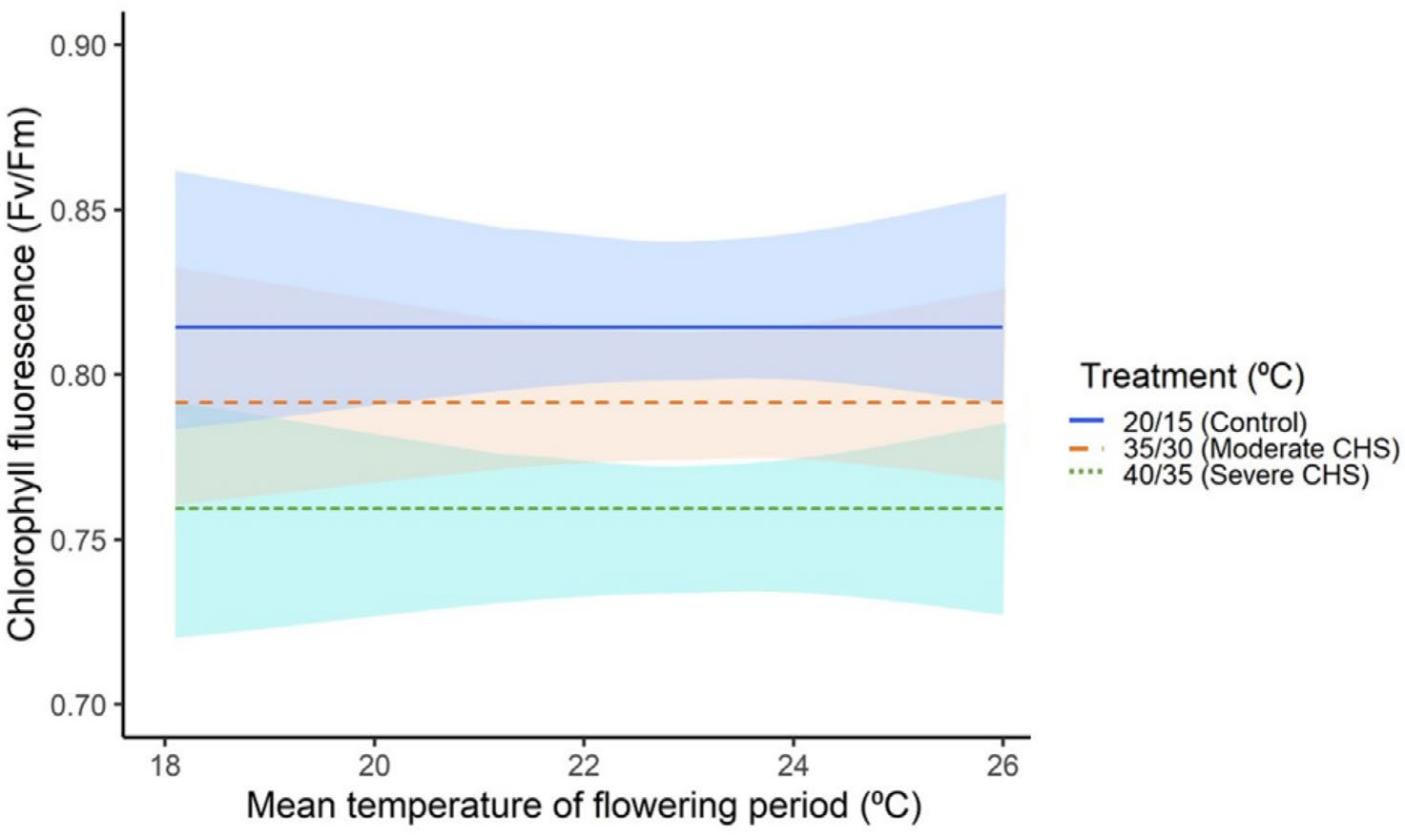
Variation in chlorophyll fluorescence (*Fv/Fm*) values in 11 *Silene vulgaris* populations under two chronic heat stress treatments (moderate and severe), along with a control group, across the mean temperature of the flowering period. Shaded areas indicate a 95% confidence interval.

The application of both CHS treatments had a significant negative impact on the overall vegetative plant performance regardless of plant origin, with the most pronounced effects in severe CHS treatment (mean *Fv*/*fm* values: control = 0.82, moderate CHS = 0.795, and severe CHS = 0.77; Figure 2, Table 2).

**TABLE 2:** The adaptation (slope of the control regression) and acclimation potential (overall treatment effects and regression slopes) of traits measured (leaf chlorophyll fluorescence [*Fv*/*fm*], female and male gametophyte, and seed traits) in the 11 wild *Silene vulgaris* populations. Results determined from analyses employing generalized linear mixed‐effect models and post hoc Tukey tests. Significance, denoted by bold formatting, is attributed to treatment/slope effects (*p* < 0.05). Distinctions between the control and both treatments, as discerned through the Tukey post hoc test (*p* < 0.05), are represented by dissimilar letters. SE refers to standard error.

Measured traits	Estimates	Control	35°C/30°C	40°C/35°C
*Fv*/*fm*	Mean	0.82 a	**0.795** b	**0.774** c
	SE±	0.03	0.04	0.05
	Slope	−0.002	0.0025	0.0026
Ovary length (mm)	Mean	3.64 a	**3.91**b	3.70 ab
	SE±	0.89	0.87	1.23
	Slope	−0.023	−0.037	−0.061
Ovule count	Mean	88 a	94 a	**66** b
	SE±	33.09	32.40	38.95
	Slope	1.32	−0.87	−2.49
Ovule size (mm)	Mean	0.27 a	**0.31** b	**0.29** bc
	SE±	0.08	0.09	0.12
	Slope	−0.004	−0.0065	−0.0029
Anther length (mm)	Mean	2.5 a	**2.2** b	**2.0** c
	SE±	0.66	0.56	0.87
	Slope	0.0157	0.0433	−0.0613
Pollen count	Mean	2522 a	**1315** b	**649** c
	SE±	878.34	832.31	583.58
	Slope	9.35	−5.62	−20.13
Pollen size (mm)	Mean	0.043 a	**0.036** b	**0.033** c
	SE±	0.01	0.01	0.01
	Slope	4.01E‐05	−3.17E‐04	**0.0015**
Seed number	Mean	221 a	**13** b	**56** c
	SE±	329.54	17.12	67.63
	Slope	**−73.55**	−4.88	−17.51
Seed mass (g)	Mean	0.3 a	**0.02** b	**0.07** c
	SE±	0.55	0.03	0.10
	Slope	**−0.123**	−0.0067	−0.026

### 3.2 Female Gametophytic Traits

**FIGURE 3:**
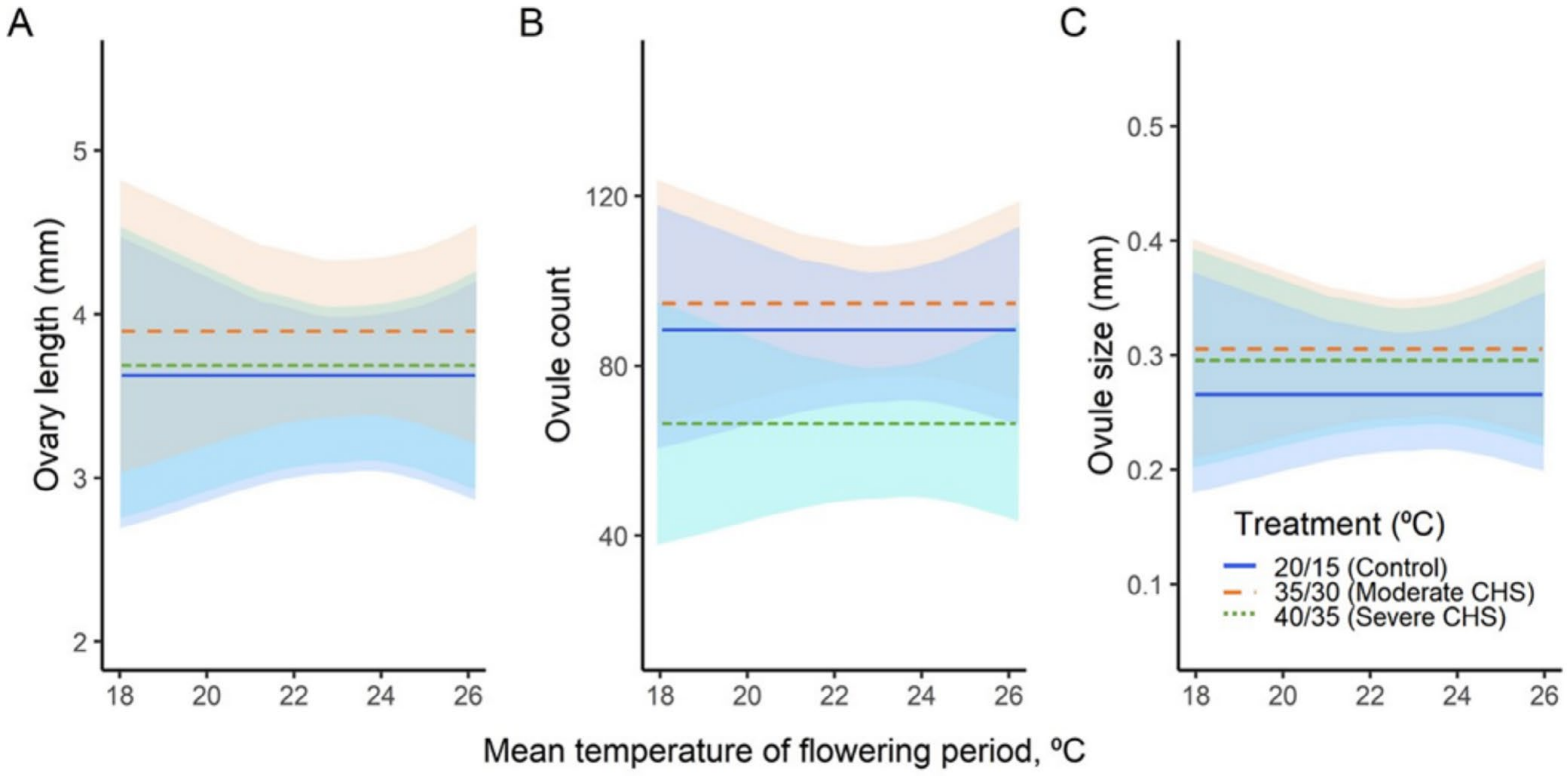
Variations of ovary length (A), ovule production (B), and ovule size (C) in 11 *Silene vulgaris* populations under two chronic heat stress treatments (moderate and severe), along with a control group, across the mean temperature of the flowering period. Shaded areas indicate a 95% confidence interval. Regression lines parallel to the *x*‐axis indicate a lack of statistical differences among the study populations.

In the common garden experiment (*H*_1_), there were no differences in female gametophytic traits (ovary length, ovule production, and ovule size) in plants from different climates across the temperature gradient, as the slopes of the corresponding regression lines were not significantly different from zero (Figure 3, Table 2).

When exposed to moderate CHS treatments (*H*_2_), plants produced significantly increased ovary length (3.91 mm) and larger‐sized ovules (0.31 mm) compared to the control (3.64 and 0.27 mm, respectively; Figure 3A,C; Table 2).

In response to severe CHS treatment (*H*_*3*_), plants exhibited a significant decrease in ovule production (66 ovules), in contrast to both moderate CHS treatment (94) and the control (88). Contrary to this, ovule size in the plants significantly increased, as shown by their average ovule size of 0.29 mm compared to the control (0.27 mm; Figure 3B,C; Table 2).

In both moderate and severe CHS treatments, the female gametophytic traits in plants from different climates showed no significant differences along the temperature gradient, as the slopes of the corresponding regression lines were not significantly different from zero (Figure 3, Table 2).

### 3.3 Male Gametophytic Traits

**FIGURE 4:**
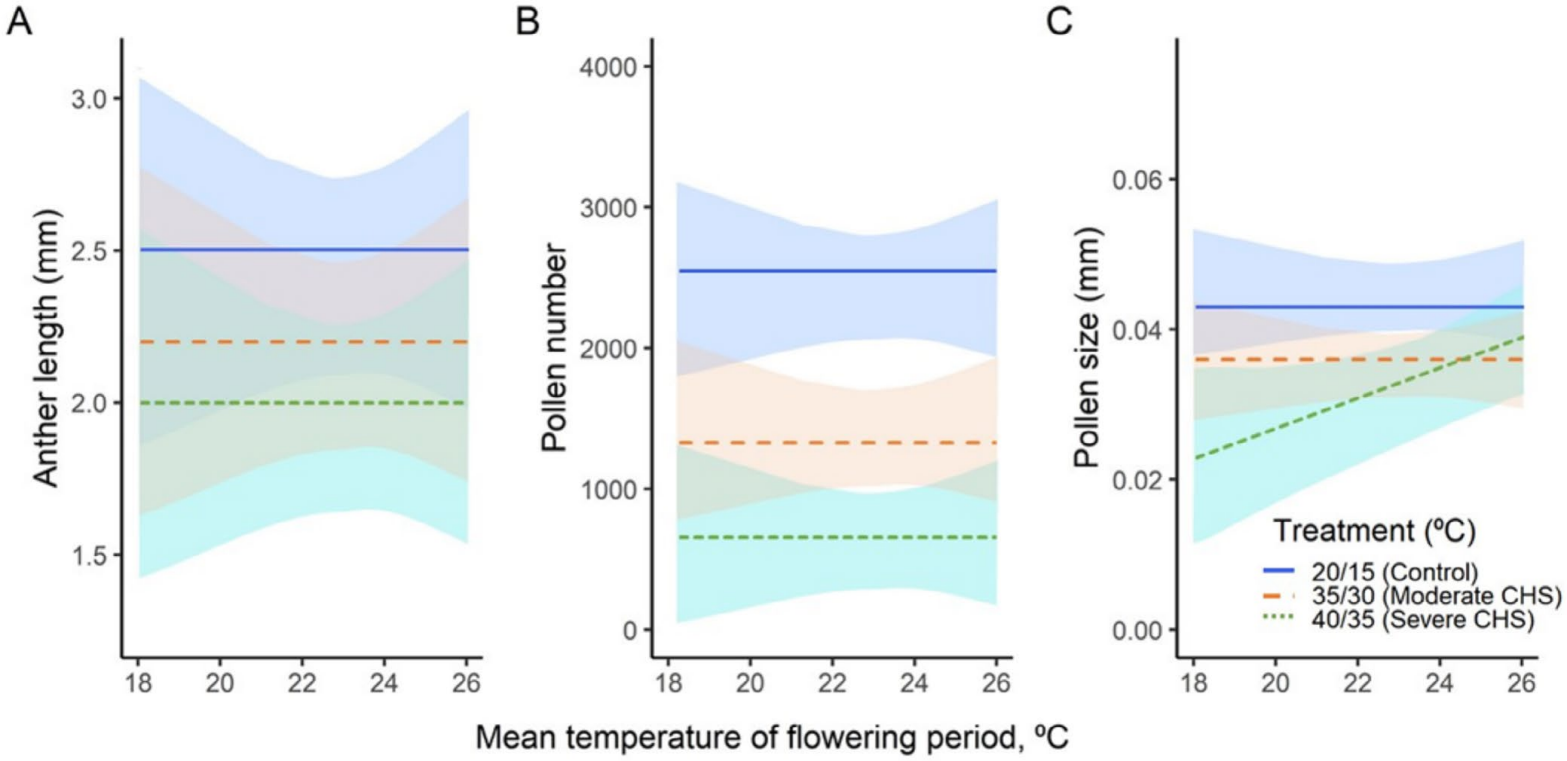
Variations of anther length (A), pollen production (B), and pollen size (C) in 11 *Silene vulgaris* populations under two chronic heat stress treatments (moderate and severe), along with a control group, across mean temperature of the flowering period. Shaded areas indicate a 95% confidence interval. Regression lines parallel to the *x*‐axis indicate a lack of statistical differences among the study populations.

In the common garden (*H*_1_), plants originating from both warm and cold climates showed no differences in male gametophytic traits along the temperature gradient, as the slopes of the corresponding regression lines were not significantly different from zero (Figure 4, Table 2).

All three male gametophytic traits measured showed a significant negative response to both heat stress treatments, with significantly larger effect sizes in the severe CHS treatment (*H*_3_) compared to the moderate CHS (*H*_2_) and the control (Figure 4, Table 2). Specifically, in moderate CHS, plants showed significantly reduced anther length (2.2 mm), pollen production (1315), and pollen size (0.036 mm) compared to the control (2.5 mm, 2522 pollen, and 0.043 mm, respectively).

Under severe stress conditions (*H*_3_), the male traits further significantly reduced, with the mean anther length decreasing to 2.0 mm, pollen production reducing to 649, and pollen size decreasing to 0.033 mm, in contrast to the control values of 2.5 mm, 2522 pollen, and 0.043 mm, respectively.

Plants originating from different climates did not show any significant differences in both CHS treatments along the temperature gradient, except for pollen size, which exhibited a significant positive correlation with the temperature gradient under severe CHS treatment (*p* < 0.05; Figure 4C, Table 2).

Plants from the warmest climates exhibited larger pollen sizes, while those adapted to colder climates had smaller pollen sizes. Across all plant origins, a 0.0015 mm reduction in pollen size per 1°C decrease in average temperature of the flowering period was found under severe CHS.

### 3.4 Seed Traits

**FIGURE 5:**
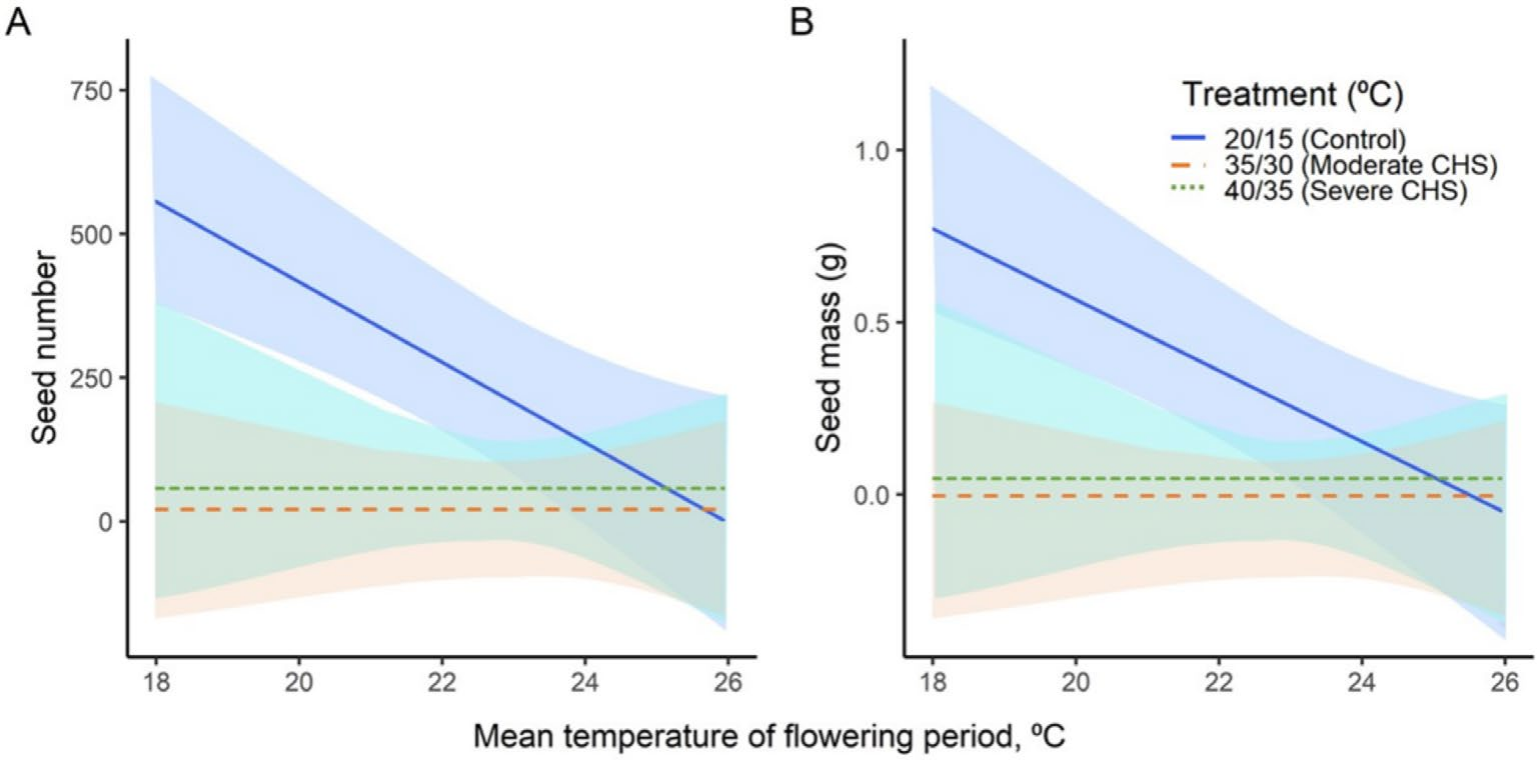
Variations in seed production (A) and seed mass (B) in 11 *Silene vulgaris* populations under two chronic heat stress treatments (moderate and severe), along with a control group, across mean temperature of flowering period. Shaded areas indicate a 95% confidence interval. Regression lines parallel to the *x*‐axis indicate a lack of statistical differences among the study populations.

In the common garden, plants from warmer climates exhibited a significant negative decline in seed production and mass, with a reduction of 74 seeds and 0.1 g (seed mass) per degree of increasing temperature. Conversely, plants originating from colder climates tend to yield more larger seeds (*p* < 0.05; Figure 5, Table 2).

Both CHS treatments applied to the plants resulted in a significant decrease in both mean seed production and seed mass compared to the control group. Specifically, the plants subjected to moderate CHS treatment yielded 13 seeds with a seed mass of 0.02 g, whereas those exposed to severe CHS treatment produced 56 seeds with a seed mass of 0.07 g, in contrast to the control which produced 221 seeds with a seed mass of 0.3 g (Figure 5, Table 2).

Importantly, the resulting seed production and mass from plants across different populations exposed to both CHS treatments showed no differences across the temperature gradient, as the slopes of the corresponding regression lines were not significantly different from zero (Figure 5, Table 2).

Notably, warm‐climate populations, characterized by their tendency to yield small seeds in smaller quantities, experienced less pronounced negative impacts on seed production and mass under high temperatures compared to plants in regions with lower temperatures.

## 4 Discussion

### 4.1 Common Garden Experiment Reveals No Adaptation in Gametophytic Traits to Local Growing Conditions (H_1_ )

Contrary to our expectations, we found no significant differences in all male and female gametophytic traits measured across the temperature gradient in the common garden experiment. This finding suggests that these traits in *Silene vulgaris* do not play any role in the plant reproductive adaptation to the specific conditions of their local origins. This contradicts the commonly observed variation in other traits such as vegetative traits like canopy height (Jónsdóttir et al. 2023) and specific leaf area (Rosbakh et al. 2015). While research indicates that numerous plant species exhibit local adaptation, some argue that local adaptation might be less prevalent than commonly thought (e.g., Hereford 2009). For instance, a study by Ebeling et al. (2011) on *Buddleja davidii* , an ornamental shrub, found no evidence of clinal variation in growth and reproductive traits among the different populations.

The lack of clinal variation observed in the gametophytic traits assessed in our study could be attributed to several factors. Firstly, rather than altering the size and number of anthers, ovaries, pollen grains, and ovules, plants may adapt the physiology and biochemistry of these organs. For example, previous studies have shown that warm‐adapted pollen tends to have higher concentrations of sugars such as glucose and fructose, more unsaturated fatty acids in lipids, and increased expression of heat‐shock proteins and antioxidants to counteract heat stress (Nievola et al. 2017; Rieu et al. 2017). On the other hand, cold‐adapted pollen tends to have lower sugar levels, increased proportions of saturated fatty acids, and synthesizes cold shock proteins or antifreeze proteins to cope with low temperatures (Satyakam et al. 2022; Jahed et al. 2023). Consequently, these adjustments rarely influence the morphological characteristics of both male and female gametophytes, as could be the case in our study. Secondly, plants possess the ability to adjust their phenological timing to optimize resource utilization under local temperature conditions, thereby minimizing variations in gametophytic traits. For instance, a study on *Arabidopsis lyrata* revealed that southern populations flowered earlier than their northern counterparts (Riihimäki and Savolainen 2004). Similarly, in our study, populations from southern Europe (e.g., Spain) exhibited early flowering, whereas northern populations (e.g., Sweden) flowered later (see Table 1). This difference could be a strategy for southern populations to evade heat stress associated with high temperatures in later months, while the northern populations may benefit from warmer periods (e.g., Rauschkolb et al. 2023). If plants from diverse locations have adapted to distinct phenological timings in their native environments, it suggests that their reproductive processes are already adjusted to their local conditions. Lastly, the gametophytic traits might be phylogenetically constrained, i.e., evolutionarily conserved across related species, and thus are not subject to intraspecific variation (e.g., Emilio et al. 2021).

Although gametophytic traits did not exhibit clinal variation, significant differences were observed in seed production and mass along the temperature gradient (Figure 5; Table 2). Plants originating from colder climates produced more seeds of greater mass compared to those from warmer climates. This could be attributed to the influence of priority sinks on plant growth patterns, with seeds having the highest sink strength (Wardlaw 1990; Obeso 2002). In colder climates, where growth and survival conditions are less favorable, plants tend to allocate more resources toward seed production as an adaptation strategy. This results in the production of larger seeds for better establishment in the unfavorable climatic conditions (Moles and Westoby 2006; Zhou et al. 2021; Celebias and Bogdziewicz 2023). On the contrary, within warmer southern populations, plants tend to produce smaller seeds as an adaptation to cope with the stress of higher temperatures (e.g., Zhou et al. 2021). This occurs especially as they approach the edge of their distribution range, where plants are near their tolerance limits. Consequently, these plants allocate fewer resources to seed production, prioritizing other survival mechanisms like efficient water use and growth (e.g., Huot et al. 2014; Lauder et al. 2019; Zhou et al. 2021).

### 4.2 The Acclimation Potential of Gametophytic Traits

#### 4.2.1 Overall Treatment Effects

Acclimation plays a pivotal role in enabling plants to enhance their tolerance to environmental extremes by directly modifying their physiology or morphology (Sumner et al. 2022). The gametophytes of *Silene vulgaris* in our study demonstrated the capacity for acclimation.

Female gametophytes exposed to moderate heat stress (*H*_2_) showed larger ovaries and produced a larger number of larger ovules, whereas male gametophytes produced fewer and smaller pollen grains, with shorter anthers (Figures 3 and 4). The longer ovaries likely provide more space for ovules to develop, and larger ovules may contain more resources, potentially enhancing support for embryo development and increasing the likelihood of viable seed production (e.g., Strelin and Aizen 2018; Wilkinson et al. 2019). The observed higher ability of female gametophytic traits to acclimate suggests potential trade‐offs between female and male traits, where more resources are allocated to female reproductive structures to mitigate the adverse effects of the heat stress on male gametophytes (Gillet and Gregorius 2020). Moreover, heat stress appeared to directly hinder physiological processes critical for optimal male gametophyte development, leading to shorter anthers that produced fewer and smaller pollen grains compared to the control group (Figure 4; Hasanuzzaman et al. 2013; Kumar et al. 2022). Consequently, the reduced number and size of pollen grains decreased the likelihood of successful pollination and fertilization, ultimately resulting in lower seed quantity and quality (Figure 5; Huang et al. 2014; Tushabe et al. 2023).

Averaged over all populations, under severe stress conditions (*H*_3_), fewer but large‐sized ovules, less pollen of smaller sizes, and shorter anthers were produced. These results confirm our hypothesis and likely stem from adjustments in resource allocation to withstand the stress (Ruan et al. 2013; Brock et al. 2017). The fewer yet larger ovules indicate a shift in resource allocation towards producing fewer but potentially more resilient or viable ovules. This reallocation suggests that larger ovules have a higher chance of survival or successful fertilization in stressful environments (Gillet and Gregorius 2020). Conversely, the decrease in pollen production, smaller pollen size, and shorter anther length indicate a reduced investment in male gametophytic structures, likely due to resource scarcity and physiological constraints imposed by severe stress, hindering pollen formation and viability (Müller and Rieu 2016; Gillet and Gregorius 2020; Chaturvedi et al. 2021).

Our study further found that the gametophytic acclimation responses were not effective in coping with both CHS treatments, resulting in a significant decrease in both seed size and number (Table 2; Figure 5). It is also possible that the heat treatments were too intense for these adaptation mechanisms to counteract the stress (e.g., Huang et al. 2014; Djanaguiraman et al. 2018). Moreover, the lowered overall performance of the plants, as evidenced by decreased rates of photosynthesis in both heat treatment conditions (Figure 2), likely contributed to the reduced seed production, as the plants' capacity to generate and store the essential resources vital for seed formation experienced a decline (see, e.g., Sommer et al. 2023). Under high temperature stress, plants may allocate more resources towards growth or survival traits rather than reproduction, potentially enhancing plant fitness (Sakata et al. 2010; Huot et al. 2014; Sharma et al. 2018). However, the opposite strategy has also been observed in nature, where some plants increase seed production under stressful conditions (Zhang et al. 2020).

#### 4.2.2 Acclimation Potential of Gametophytic Traits Along the Temperature Gradient

Contrary to our expectations, all plants from different climates did not show any differences in their acclimation potential to both CHS treatments along the temperature gradient, except for pollen size under severe CHS treatment (Figure 4C). Specifically, plants from warmer climates showed a greater acclimation ability, with larger pollen size at higher temperatures compared to those from colder areas. We explain this finding by the fact that high temperatures are associated with increased evapotranspiration rates, which can rapidly desiccate pollen grains, reducing their viability and thus affecting successful pollination. Therefore, the optimal strategy for plants flowering under high temperatures is to produce fewer but larger pollen grains that are more resilient to volume changes (Ejsmond et al. 2011).

The lack of differences in acclimation potential for the other traits across the temperature gradient may be due to similar factors explained in the common garden experiment (see above). They include plants prioritizing adaptations in gametophyte physiology and biochemistry over morphological changes in size and quantity (e.g., Nievola et al. 2017; Rieu et al. 2017) or adjusting their phenology timings to optimize resource utilization and enhance survival in diverse environments (e.g., Cook et al. 2012; Gugger et al. 2015). Additionally, gametophytic traits might be phylogenetically constrained, thus limiting variations across climatic gradients (e.g., Emilio et al. 2021).

## 5 Conclusions

The evaluation of the male (anther length, pollen production, and size) and female (ovary length, ovule production, and size) gametophytic traits of wild *Silene vulgaris* populations in response to heat stress revealed a lack of adaptation and/or acclimation mechanisms (such as morphological adjustments) along the temperature gradient. This prompts the question of how gametophytes in natural plant populations, especially in southern regions, cope with heat stress. One plausible explanation is the presence of alternative mechanisms or adaptation strategies, such as alterations in flowering phenology, enabling survival without specific gametophytic adaptations. However, these strategies have limitations due to physiological and, possibly, genetic constraints. For instance, inconsistencies in environmental cues for flowering or sudden environmental changes can disrupt flowering timing, leading to mismatches with optimal gametophytic performance affecting reproductive success (e.g., Fu et al. 2022; Elmendorf and Hollister 2023). The negative effects on gametophyte performance can then be translated to reduced seed numbers and quality, also observed in this study. Producing fewer seeds can lead to reduced dispersal, germination success, and adaptation abilities for plants (Jakobsson and Eriksson 2000; Soons and Heil 2002; Long et al. 2015). This could result in smaller, less diverse populations, making species more vulnerable to extinction due to limited abilities to adapt to changing conditions (Long et al. 2015; Schierenbeck 2017). Consequently, further research into alternative adaptation strategies and mechanisms, including phenotypic plasticity, which was not accounted for in this study, is crucial for gaining insights into the resilience of natural plant populations in the face of ongoing climate change.

## Author Contributions

**Donam Tushabe:** formal analysis (lead), investigation (equal), methodology (equal), project administration (equal), supervision (supporting), writing – original draft (lead), writing – review and editing (lead). **Franziska Altmann:** data curation (equal), investigation (equal), methodology (equal), writing – review and editing (equal). **Erik Koehler:** data curation (equal), investigation (equal), methodology (equal), writing – review and editing (equal). **Sebastian Woods:** data curation (equal), methodology (equal), project administration (equal), resources (supporting), writing – review and editing (equal). **Sandra Kahl:** data curation (equal), methodology (equal), resources (equal), writing – review and editing (equal). **Sergey Rosbakh:** conceptualization (lead), data curation (equal), formal analysis (equal), funding acquisition (lead), methodology (equal), project administration (lead), resources (equal), supervision (lead), writing – original draft (equal), writing – review and editing (equal).

## Conflicts of Interest

The authors declare no conflicts of interest.

## Supporting information


Data S1

## Data Availability

The original contributions presented in the study are included in the Supporting Information for the article; further inquiries can be directed to the corresponding author.
