# Supplementary material for: Adaptation and Acclimation of Gametophytic Traits to Heat Stress in a Widely Distributed Wild Plant Along a Steep Climatic Gradient
Source: Ecol Evol. 2025 Mar 30;15(4):e71199. doi: 10.1002/ece3.71199 (PMC11955256; doi:10.1002/ece3.71199)
Supplement: Supplementary file 1 — Data S1 [file ECE3-15-e71199-s001.docx]

# Supplementary material


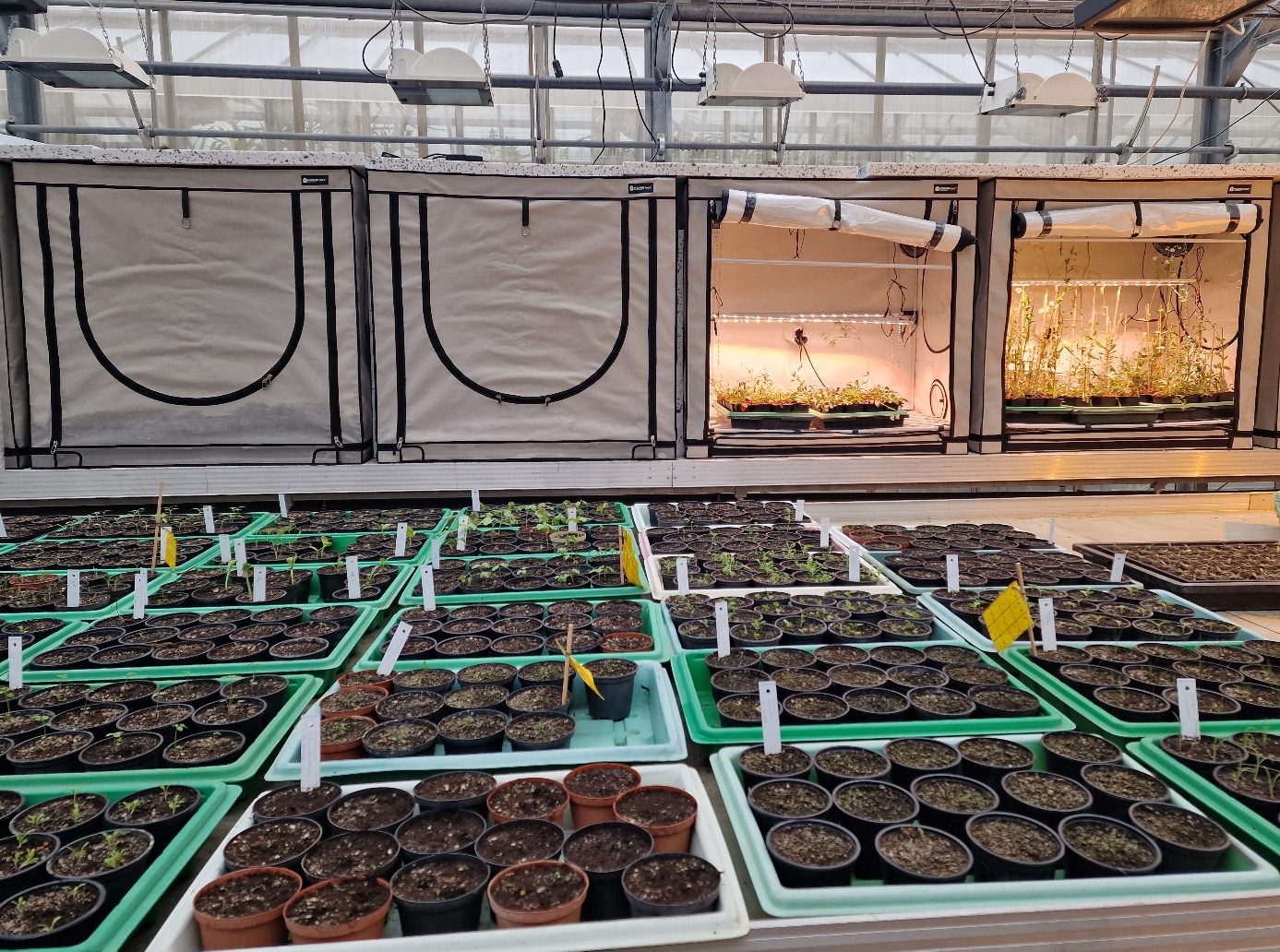


Figure S1: Grow chambers used for the experiment in the greenhouse


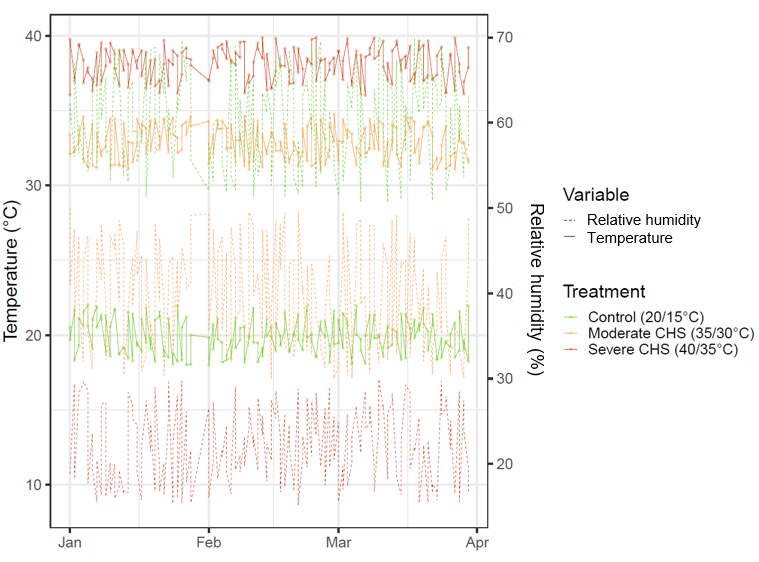


Figure S2: Temperature and relative humidity under control and chronic heat stress treatment conditions during the experiment for the populations of *Silene vulgaris*
